# Allosteric Modulators of Sigma-1 Receptor: A Review

**DOI:** 10.3389/fphar.2019.00223

**Published:** 2019-03-19

**Authors:** Edijs Vavers, Liga Zvejniece, Tangui Maurice, Maija Dambrova

**Affiliations:** ^1^Laboratory of Pharmaceutical Pharmacology, Latvian Institute of Organic Synthesis, Riga, Latvia; ^2^MMDN, University of Montpellier, INSERM, EPHE, UMR-S1198, Montpellier, France

**Keywords:** sigma-1 receptor (Sig1R), allosteric modulator, phenytoin, E1R, SOMCL-668, SKF83959, OZP002, fenfluramine

## Abstract

Allosteric modulators of sigma-1 receptor (Sig1R) are described as compounds that can increase the activity of some Sig1R ligands that compete with (+)-pentazocine, one of the classic prototypical ligands that binds to the orthosteric Sig1R binding site. Sig1R is an endoplasmic reticulum membrane protein that, in addition to its promiscuous high-affinity ligand binding, has been shown to have chaperone activity. Different experimental approaches have been used to describe and validate the activity of allosteric modulators of Sig1R. Sig1R-modulatory activity was first found for phenytoin, an anticonvulsant drug that primarily acts by blocking the voltage-gated sodium channels. Accumulating evidence suggests that allosteric Sig1R modulators affect processes involved in the pathophysiology of depression, memory and cognition disorders as well as convulsions. This review will focus on the description of selective and non-selective allosteric modulators of Sig1R, including molecular structure properties and pharmacological activity both *in vitro* and *in vivo*, with the aim of providing the latest overview from compound discovery approaches to eventual clinical applications. In this review, the possible mechanisms of action will be discussed, and future challenges in the development of novel compounds will be addressed.

## Introduction

The International Union of Basic and Clinical Pharmacology included sigma receptor in its list of receptors only in 2013 as a ligand-regulated non-opioid intracellular receptor (Alexander et al., 2013). Two pharmacologically distinct subtypes of sigma receptor, namely, sigma-1 receptor (Sig1R) and sigma-2 receptor (Sig2R), have been identified (Hellewell and Bowen, 1990; Quirion et al., 1992; Hellewell et al., 1994). Sig2R is known also as endoplasmic reticulum-resident transmembrane protein TMEM97 (Alon et al., 2017) involved in the regulation of cholesterol homeostasis and cell differentiation (Bartz et al., 2009; Haller et al., 2012). Number of selective Sig1R and Sig2R ligands have been described confirming significant differences in the pharmacological regulation of these subtypes. Thus, far no allosteric modulators of Sig2R have been reported.

Sig1R is an integral membrane-bound protein that is found in the nuclear membrane and endoplasmic reticulum and mitochondria-associated membrane (Mori et al., 2013; Mavlyutov et al., 2015; Su et al., 2016). Sig1R is expressed in both the CNS and peripheral tissues (Su and Junien, 1994). Sig1R is widely distributed in the brain, and it concentrates in specific areas involved in memory, emotion and sensory and motor functions (Alonso et al., 2000; Cobos et al., 2008). Sig1R, as described by its functional nature, is a chaperone protein and a unique cell protein modulator [reviewed in (Su et al., 2016)] that can amplify or reduce the signaling initiated when interacting with target proteins (Hayashi and Su, 2007; Zamanillo et al., 2012; Rodríguez-Muñoz et al., 2015). Therefore, Sig1R demonstrates properties that can be attributed to both chaperone proteins and receptors. However, the notion that allosteric modulators of Sig1R are identified is an additional argument for the “receptor” view of Sig1R.

**Figure 1:**
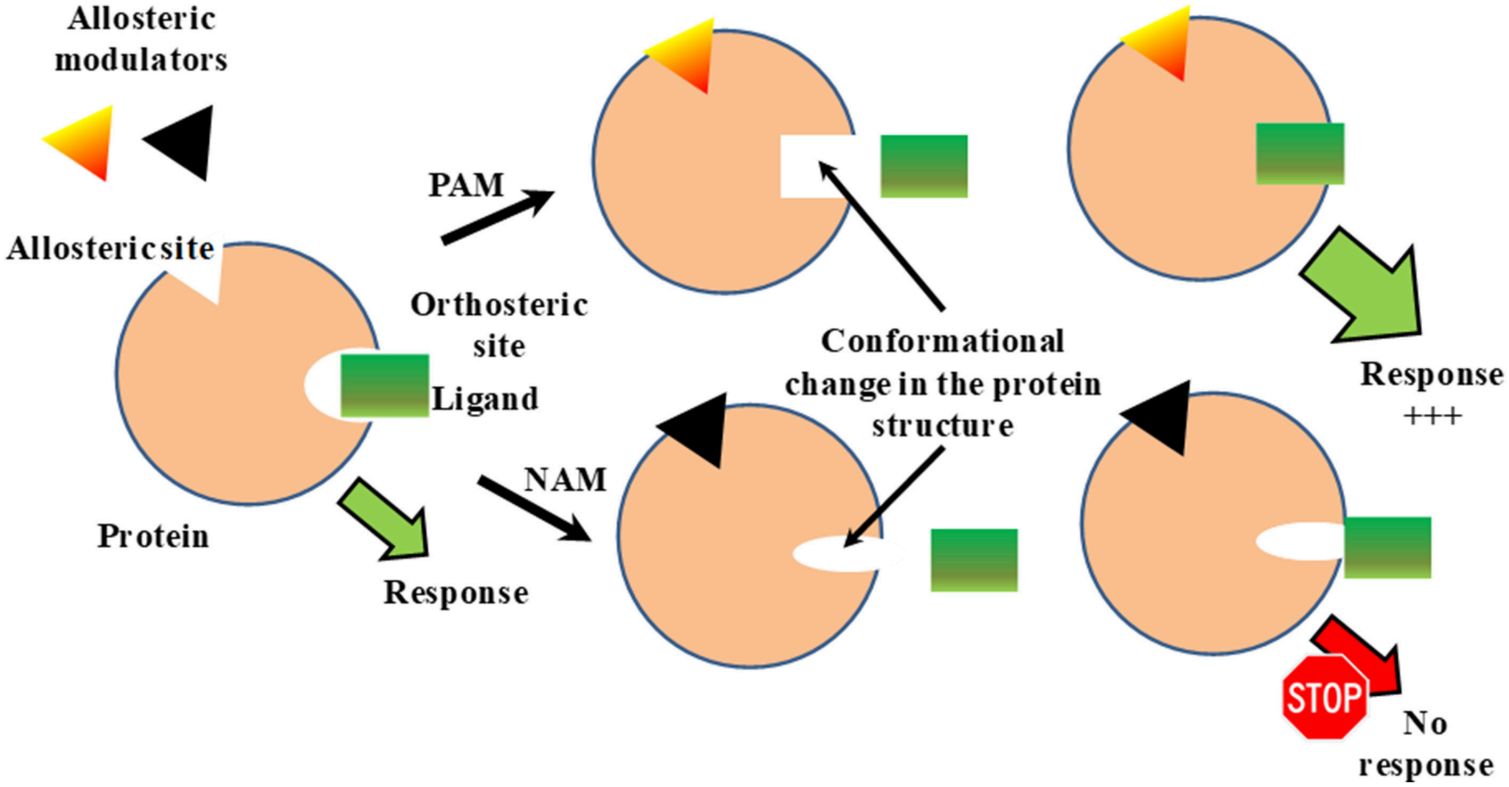
Classical model for allosteric regulation of receptor. PAM, positive allosteric modulation; NAM, negative allosteric modulation. This Figure has been modified from Vavers (2017).

Allosteric regulation is the regulation of protein activity by binding an effector molecule at a site other than the orthosteric or active site of a protein (Figure 1). The binding of allosteric modulators to a target protein induces a conformational change in the protein structure and changes the activity of orthosteric ligands (Figure 1). Allosteric modulators can be positive or negative effectors (PAMs or NAMs, respectively). PAMs increase the activity of the ligand, while NAMs block it (Figure 1). To date phenytoin, some benzazepine derivatives and stereoisomers of methylphenylpiracetam have been reported as PAMs of Sig1R while NAMs of Sig1R have not yet been described. The definition of allosteric Sig1R modulators might be artificial due to a lack of information on the orthosteric binding site for Sig1R. It is thought that the orthosteric binding site for Sig1R ligands is the binding site of (+)-SKF-10,047 and (+)-pentazocine, benzomorphan compounds that were the first identified to bind to Sig1R with high affinity and selectivity (Martin et al., 1976; Su, 1982). Thus, allosteric modulators of Sig1R are described as compounds that can increase the activity of Sig1R ligands that compete with [^3^H](+)-pentazocine for binding to Sig1R.

At the moment, two compounds, the Sig1R and muscarinic receptor mixed agonist ANAVEX™ 2-73 (ANAVEX Life Sciences, ClinicalTrials.gov Identifier: NCT02244541) and the Sig1R antagonist E-52862 (ESTEVE, EudraCT number: 2012-000400-14) are being tested in clinical trials for the treatment of Alzheimer's disease and neuropathic pain, respectively, thus justifying the importance of Sig1R as a valid molecular target for clinical applications. Compared to widely used agonists and antagonists, allosteric modulators are less studied but are highly promising due to their advantageous clinical applications.

In recent years, significant progress has been achieved in the discovery, optimization and preclinical development of allosteric Sig1R modulators. These compounds can provide new advances in developing novel drugs, drug leads and research tools for Sig1R and have potential utility for the treatment of multiple human disorders. Accumulating evidence suggests that allosteric Sig1R modulators affect processes involved in the pathophysiology of depression, memory and cognition disorders as well as convulsions, and thus can provide novel strategies for the treatment of neurological disorders.

This review summarizes the literature and data on all known Sig1R allosteric modulators, including discovery and development, *in vitro* and *in vivo* pharmacological activities and discussion on allosteric regulation of Sig1R.

## Discovery of Allosteric Sig1R Modulators

**Figure 2:**
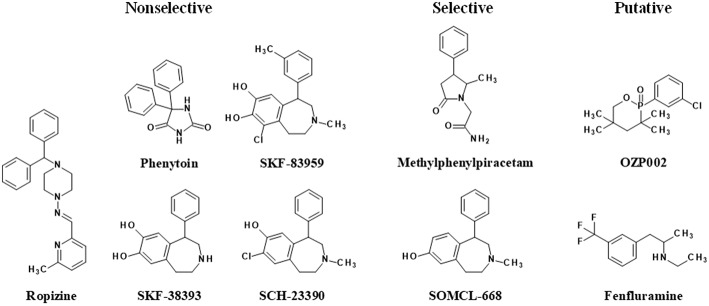
Allosteric modulators of Sig1R. Allostericity was not yet demonstrated for OZP002 and fenfluramine, only the Sig1R modulatory effect, and must therefore be considered as putative PAMs.

The first evidence indicating that a compound demonstrates allosteric activity on Sig1R came from radioligand binding studies. The first drug discovered as an allosteric modulator of Sig1R was phenytoin (diphenylhydantoin, Figure 2). Phenytoin has been used in clinical practice as an anti-convulsant since 1930 (Merritt and Putnam, 1984; Yaari et al., 1986). The anti-convulsant mechanism of phenytoin is the selective blockage of neuronal voltage-dependent sodium channels (Yaari et al., 1986). Over the course of competition binding studies, it was shown that phenytoin can increase the binding of [^3^H]dextromethorphan ([^3^H]DM) (Craviso and Musacchio, 1983) and [^3^H](+)-3-(3-hydroxyphenyl)-N-propylpiperidine ([^3^H](+)-3-PPP) in the guinea pig brain (Musacchio et al., 1989). These results were the first showing that phenytoin allosterically modulated the binding of prototypic sigma site ligands. Very rapidly, phenytoin sensitivity was even considered an intrinsic characteristic of the sigma-1 subtype of sigma sites, not shared by sigma-2 (Quirion et al., 1992). Sig1R were indeed defined mainly through their high-affinity sites for the dextrorotatory isomers of benzomorphans and their sensitivity to phenytoin. Moreover, similar to phenytoin, ropizine (SC-13504), an anti-convulsant benzhydryl piperazine (Figure 2), induced a marked concentration-dependent increase in the binding of [^3^H]DM (Musacchio et al., 1988) and [^3^H](+)-3-PPP (Musacchio et al., 1989). It was shown that the non-narcotic anti-tussive noscapine can dose-dependently potentiate the binding of [^3^H]DM in guinea pig brainstem homogenate (Craviso and Musacchio, 1983). In addition, hydrastine demonstrated similar activity on the binding of [^3^H]DM (Craviso and Musacchio, 1983). However, to date, noscapine and hydrastine have not been demonstrated to modulate the binding of more selective Sig1R ligands such as [^3^H](+)-pentazocine and are considered only putative allosteric Sig1R modulators.

Later, compound SR31747A (N-cyclohexyl-N-ethyl-3-(3-chloro-4-cyclohexylphenyl)propen-2-ylamine hydrochloride) was also proposed to act as an allosteric modulator of peripheral sigma binding sites (Paul et al., 1994). Although SR31747A modulated the activity of Sig1R ligands *in vivo* and *in vitro*, the use of radiolabeled [^3^H]SR31747A demonstrated that SR31747A binds specifically, saturably and reversibly to rat spleen membranes and human lymphocytes at a single class of high-affinity sites, which were clearly different from the [^3^H](+)-pentazocine and [^3^H](+)-3-PPP binding sites (Paul et al., 1994). A few years later, the purified amino acid sequence of the [^3^H]SR31747A binding site was found to be a nuclear membrane protein related to a fungal C8-C7 sterol isomerase; additionally, this protein, which was called SR31747A-binding protein, was encoded by the ERG2 gene (Jbilo et al., 1997). The exact molecular mechanism of SR31747A has not been fully described thus far, but SR31747A is currently not considered an allosteric Sig1R modulator.

In 2013, more than two decades after the discovery of the positive allosteric Sig1R modulatory activity of phenytoin, it was shown that the atypical dopamine D_1_ receptor agonist SKF83959 (3-methyl-6-chloro-7,8-hydroxy-1-[3-methylphenyl]-2,3,4,5-tetrahydro-1H-3-benzazepine) and its analogs SKF38393 and SCH23390 act as allosteric Sig1R modulators (Guo et al., 2013; Figure 2). These compounds could enhance the binding activity of [^3^H](+)-pentazocine in brain and/or liver tissues, shift the saturation curve toward the left, and decrease the dissociation rate in binding kinetic analysis (Guo et al., 2013). At the same time another allosteric Sig1R modulator, methylphenylpiracetam (2-(5-methyl-2-oxo-4-phenyl-pyrrolidin-1-yl)-acetamide), was described (Veinberg et al., 2013; Figure 2). The activity of E1R, a 4R,5S-isomer of methylphenylpiracetam, was profiled using commercially available screening assays, which showed that the compound selectively modulates Sig1R activity but does not affect the other investigated neuronal receptors and ion channels (Zvejniece et al., 2014). The following detailed *in vitro* studies, in which more selective Sig1R ligands were used, provided solid evidence that E1R is a PAM of Sig1R (Zvejniece et al., 2014). Sig1R is the only molecular target described thus far that accounts for the pharmacological activity of E1R. Therefore, E1R is considered the first known selective allosteric Sig1R modulator. Later, several chemical derivatives of SKF83959 were synthesized to find a selective Sig1R allosteric modulator and to exclude the potential involvement of other receptors. One of these newly synthesized compounds, called SOMCL-668 (Figure 2), did not exhibit affinity for human dopamine D_1_, D_2_, D_3_, serotonin 5-HT_1A_, or 5-HT_2A_ receptors (Zhang et al., 2014) but did show potent allosteric modulating activity at Sig1R (Guo et al., 2015). Therefore, SOMCL-668 has also been proposed as a selective allosteric Sig1R modulator.

Recently, a novel oxazaphosphinane compound derived from hydroxybupropion, was described (Volle et al., 2010). (±)-2-(3-chlorophenyl)-3,3,5,5-tetramethyl-2-oxo-[1,4,2]-oxazaphosphinane (OZP002) did not inhibit [^3^H](+)-pentazocine binding to Sig1R, as did bupropion or hydroxybupropion (Ritz and George, 1997), but rather moderately increased it. However, the drug did potentiate Sig1R agonist-induced antidepressant and anti-amnesic effects in wild-type mice but not in Sig1R-knock-out animals, and the effects were prevented by a Sig1R antagonist NE-100 suggesting a Sig1R positive modulatory effect (Maurice et al., 2017). Finally, fenfluramine (N-ethyl-α-methyl-3-(trifluoromethyl)-benzeneethanamine), a potent serotonin releaser activating multiple 5-HT receptor subtypes (Fuller et al., 1988), has been described for its positive modulatory activity at Sig1R (Maurice et al., 2018). Beyond serotonin, fenfluramine also binds Sig1R with high nanomolar affinity for Sig1R. However, in functional assays, fenfluramine potentiated the (+)-SKF-10,047-induced increase in the twitch contraction amplitude and the Sig1R/binding immunoglobulin protein (BiP) dissociation induced by the Sig1R agonist PRE-084, suggesting a positive modulatory action at Sig1R (Maurice et al., 2018).

The list of identified or suspected Sig1R positive modulators is presently being extended, and they present effective pharmacological activity that is closely related *in vivo* to that of orthosteric agonists. However, the different chemical structures of the identified compounds suggest a complex mode of action and clear specificities among the drugs.

## The Pharmacological Activities of Allosteric Sig1R Modulators

### Phenytoin

The allosteric modulation of sigma recognition sites by phenytoin has been demonstrated by the ability of phenytoin to stimulate the binding of various tritiated Sig1R agonists (Musacchio et al., 1988; McCann and Su, 1991; Chaki et al., 1996; Cobos et al., 2005), to slow dissociation from sigma sites and to shift sigma sites from a low-affinity state to a high-affinity state (DeHaven-Hudkins et al., 1993). A detailed comparison of the effects of phenytoin on the binding of [^3^H](+)-pentazocine and [^3^H]NE-100 using saturation and kinetics assays showed that phenytoin acts as PAM and can negatively modulate the binding of [^3^H]NE-100 by decreasing the specific binding and increasing the dissociation rate from Sig1R (Cobos et al., 2006), demonstrating significant allosteric effects on the Sig1R ligand binding to Sig1R.

Phenytoin has been used in the clinic against various types of epileptiform seizures for more than 80 years (Santulli et al., 2016). The mechanism by which phenytoin exerts its anti-seizure activity is primarily related to the inhibition of voltage-gated sodium channels (Tunnicliff, 1996). Phenytoin has been reported to reduce increases in extracellular K^+^ concentration in neuroblastoma cells (Nobile and Lagostena, 1998) and inhibit both Na^+^ (Rush and Elliott, 1997) and T-type Ca^2+^ currents in isolated dorsal root ganglia (Todorovic and Lingle, 1998). Phenytoin was shown to block the decrease in extracellular Ca^2+^ concentration effected by repetitive stimulation of pyramidal cells in hippocampal slices (Yaari et al., 1986). In rat cerebral synaptosomes phenytoin has been shown to increase the activity of Na^+^-K^+^-ATPase (Festoff and Appel, 1968). Phenytoin is known to block Na^+^ influx through sodium channels and the binding of [^3^H]phenytoin to these channels is inhibited by sodium channel blockers (Tunnicliff, 1996). It has been shown that Sig1R is involved in the regulation of sodium channels (Johannessen et al., 2009), however it has not been clearly demonstrated that the effects of phenytoin on sodium channels are Sig1R specific, and the *in vivo* pharmacological effects of phenytoin are not Sig1R related. Therefore, phenytoin is a non-selective allosteric modulator of Sig1R.

### Ropizine (SC-13504)

Ropizine was shown to increase the binding of [^3^H]DM and [^3^H](+)-3-PPP in guinea pig brains (Musacchio et al., 1988, 1989). The allosteric effects of ropizine on [^3^H]DM binding are fully apparent at 10-fold lower concentrations than those of phenytoin (Musacchio et al., 1988). Ropizine has been shown to possess anti-convulsant activity in mice (Craig, 1967), cats (Edmonds and Stark, 1974) and dogs (Edmonds et al., 1978). It has been shown that ropizine is similar in efficacy to phenytoin in maximal electroshock-induced seizures (Novack et al., 1979), while it has limited anti-convulsant activity against chemically induced seizures (Edmonds et al., 1978, 1979). Ropizine is more active and potent than phenytoin in antagonizing the after discharges produced by cortical or hippocampal stimulation in cats (Joy and Edmonds, 1974). Data in the literature about the mechanism of action of ropizine are scarce (Table 1). It has been shown that ropizine inhibits magnesium-dependent ATPase activity in rat brain synaptosomes (Gilbert and Wyllie, 1976). Direct involvement of Sig1R in this pharmacological activity has not been demonstrated, and thus, ropizine is considered a non-selective allosteric modulator of Sig1R.

**Table 1:** Summary of *in vivo* effects of allosteric Sig1R modulators.

**Compound**	**Dose, mg/kg (route of administration)**	**Effect *in vivo***	**Animal model (species)**	**Molecular target**	**References**
Phenytoin^*^	5–20 (i.p.)	Anti-seizure activity/^*^anti-epileptic drug	Maximal electroshock-induced seizures (mice)	Voltage-gated sodium channels	Jones et al., 1981; Reviewed in Tunnicliff, 1996
	20, 40 (p.o.)	Anti-seizure activity	Ischemia-induced epilepsy (rats)		Edmonds et al., 1996
Ropizine (SC-13504)	2, 4 (i.v.)	Anti-seizure activity	Penicillin-induced epileptogenic foci (cats)	N/A	Edmonds and Stark, 1974
	4, 6 (i.v.)	Anti-seizure activity	Photosensitive epilepsy (baboons)	Magnesium-dependent ATPase	Meldrum et al., 1976
	3, 300 (p.o.)	Anti-seizure activity	Partially kindled hippocampal seizures (rats)	N/A	Albertson et al., 1978
	2.5 (ED_50_)	Anti-seizure activity	Maximal electroshock-induced seizures (rats)	N/A	Edmonds et al., 1979
SKF-83959	0.5 (i.p.)	Anti-Parkinson activity	6-OHDA-induced Parkinson's disease model (rats)	Dopamine D_1_ receptor	Zhang et al., 2007
	0.5, 1 (i.p.)		6-OHDA-induced Parkinson's disease model (rats)		Zhen et al., 2005
	2, 4, 8 (i.p.)	Anti-depressant activity	Tail suspension test; forced swimming test (mice)	SERT/NET/DAT	Fang et al., 2013
	0.5, 1 (i.p., 10 days)		Chronic social defeat stress model (mice)	Dopamine D_5_ receptor	Jiang et al., 2015
	10, 20, 40 (i.p.)	Anti-seizure activity	PTZ-induced seizures; kainic acid-induced *status epilepticus* (mice)	Sig1R	Guo et al., 2015
	1 (i.p., 11 days)	Anti-tumor activity	S.c. injection of GH3 and SCG7901 cancer cells (mice)	Dopamine D_5_ receptor	Leng et al., 2017
	0.5, 1 (i.p.)	Memory improvement	Scopolamine-induced learning deficits (mice)	Dopamine D_1_-D_2_ receptor heteromer	Sheng et al., 2018
SCH-23390	0.1–0.3 (i.p.)	Anti-seizure activity	Pilocarpine-induced seizures (rats)	Dopamine D_1_ receptor	Barone et al., 1992
	0.8 (i.p.)		Pilocarpine-induced seizures (mice)		Burke et al., 1990
	0.5 (i.p.)		Soman-induced seizures (guinea pigs)		Bourne et al., 2001
SKF-38393	1, 5, 10 (s.c.)	Anti-seizure activity	PTZ-induced seizures (mice)	Dopamine D_1_ receptor	Ogren and Pakh, 1993
	5 (i.p.)	Memory improvement	Maternal deprivation-induced memory deficiency (rats)	Dopamine D_1_ receptor	Lejeune et al., 2013
SOMCL-668	40 (i.p.)	Anti-seizure activity	PTZ-induced seizures; kainic acid-induced *status epilepticus* (mice)	Sig1R	Guo et al., 2015
	10, 20 (i.p.)	Anti-depressant activity	Forced swimming test; tail suspension test; sucrose preference test (mice)	Sig1R	Wang et al., 2016
E1R	1, 5, 10 (i.p.)	Memory improvement	Scopolamine-induced learning deficits (mice)	Sig1R	Zvejniece et al., 2014
	10, 50 (i.p.)	Anti-seizure activity	PTZ- and BIC-induced seizures (mice)	Sig1R	Vavers et al., 2017
OZP002	10, 30 (i.p.)	Anti-depressant-like activity	Forced swimming test (mice)	Sig1R	Maurice et al., 2017
	0.1, 0.3 (i.p.)	Anti-amnesic activity	Scopolamine-induced learning deficits; i.c.v. injection of amyloid Aß_25−35_ peptide; APP_Swe_ mice (mice)	Sig1R	
	0.7 (i.p.)	Neuroprotection	I.c.v. injection of amyloid Aß_25−35_ peptide (mice)	Sig1R	
Fenfluramine (ZX008)^*^	3 nmol (i.c.v.)	Anti-seizure activity	I.c.v. injection of NMDA (mice)	Serotonin 5-HT_2A_ receptor, Sig1R	Rodríguez-Muñoz et al., 2018
	0.3, 1 (i.p.)	Anti-amnesic effect	Dizocilpine-induced learning deficits (mice)	Sig1R	Maurice et al., 2018
	10, 30 (i.p.)	Anti-depressant-like activity	Forced swimming test (mice)	Serotonin 5-HT_1A_ receptor, Sig1R	Maurice et al., 2018

* Drug used in clinics;

*i.v., intravenous; i.p., intraperitoneal; s.c., subcutaneous; i.c.v., intracerebroventricular; Sig1R, sigma-1 receptor*.

### Methylphenylpiracetam (E1R)

The Sig1R site was the only site that E1R was discovered to target in *in vitro* pharmacological profiling assays, which included a number of ion channels, G protein-coupled receptors and CNS transporters (Zvejniece et al., 2014). The selected *in vitro* assays revealed that E1R did not bind directly to Sig1R but rather acted as a PAM of the receptor and enhanced the binding of an unselective sigma receptor radioligand [^3^H]DTG. E1R potentiated the contractions of rat vasa deferentia in the presence of the selective Sig1R agonist PRE-084 but not in the presence of the Sig2R agonist PB-28 (Zvejniece et al., 2014). In addition, E1R enhanced the effect of PRE-084 on the BDK-induced [Ca^2+^]_i_ increase, thus confirming its Sig1R positive allosteric modulatory effect *in vitro*.

E1R successfully alleviated scopolamine-induced cognitive impairment in mice, as assessed using passive avoidance and spontaneous alternation (Y-maze) tests. The effects of E1R were antagonized by the selective Sig1R antagonist NE-100, thus confirming the Sig1R modulatory activity of E1R *in vivo*. E1R demonstrated dose-dependent anti-convulsant effects on pentylenetetrazole (PTZ)- and bicuculline (BIC)-induced seizures at doses of 10 and 50 mg/kg (Vavers et al., 2017; Tables 1, **3**). To verify that Sig1R was involved in the anti-convulsant activity of E1R, the selective Sig1R antagonist NE-100 was used. The administration of NE-100 (5 mg/kg) before E1R (10 mg/kg) significantly restored the tonic seizure threshold to the basal level and therefore showed that the anti-seizure effect of E1R was mediated through Sig1R activity (Vavers et al., 2017). The pharmacological activity of E1R demonstrates that it is a selective PAM of Sig1R.

### Benzazepine Derivatives

#### SKF83959, SCH23390, SKF38393

SKF83959 is an atypical D_1_ agonist (Downes and Waddington, 1993; Deveney and Waddington, 1995). Previously, it was shown that SKF83959 also exerted many D_1_ receptor-independent pharmacological effects. For example, SKF83959 suppressed excitatory synaptic transmission and voltage-activated Na^+^ current in rat hippocampus (Chu et al., 2011), inhibited the delayed rectifier potassium channel in primary culture neurons (Chen et al., 2009), and promoted the spontaneous release of glutamate in the rat somatosensory cortical neurons (Chu et al., 2010). The activity of SKF83959 was related to Sig1R based on the pharmacological activity of SKF83959 and similarities of the pharmacophore with some other Sig1R ligands. SKF-83959 increased the binding of [^3^H](+)-pentazocine to Sig1R in the brain and liver tissues and showed that SKF-83959 is PAM of Sig1R (Guo et al., 2013). SKF83959 has been shown to inhibit the generation of intracellular reactive oxygen species and the expression of tumor necrosis factor α, interleukin-1β, and cytokine-inducible nitric oxide synthase in lipopolysaccharide-stimulated mouse brain microglial BV2 cells (Wu et al., 2015). These effects of SKF83959 were blocked by Sig1R antagonists BD-1047 and BD1063 (Wu et al., 2015). In the same study, it was shown that in a [^3^H](+)-pentazocine binding assay, SKF83959 enhanced the binding activity of dehydroepiandrosterone (DHEA), a neurosteroid acting as a Sig1R agonist, by shifting the DHEA binding curve to the left. In addition, SKF83959 enhanced the anti-inflammatory effect of exogenous DHEA in a synergistic manner, which was dependent on Sig1R (Wu et al., 2015), thus showing that the anti-inflammatory effects of SKF83959 are due to positive allosteric Sig1R modulator activity in cells.

The anti-convulsant effects of SKF83959 at doses of 20 and 40 mg/kg in PTZ- and kainic acid-induced seizures were demonstrated to be mediated by modulating Sig1R (Guo et al., 2015, Tables 1, **3**). The anti-convulsant effects of SKF83959 were blocked by the selective Sig1R antagonist BD-1047 at a dose of 1 mg/kg (Guo et al., 2015). SKF83959 has also demonstrated significant anti-Parkinson, anti-depressant, anti-tumor and memory-improving activity, which is associated with activity at dopamine receptors and regulation of dopamine reuptake (summarized in Table 1). In addition, it has been shown that SKF83959 inhibits voltage-gated sodium channels in cultured striatal neurons via the D_1_-like receptor-phosphatidylinositol-PKC pathway (Ma et al., 2015) and inhibits dopamine-sensitive adenylyl cyclase activity (Downes and Waddington, 1993), which are responsible for some additional *in vivo* activities not related to Sig1R.

SKF83959 analogs, such as SCH22390 and SKF38393, also have shown similar allosteric effects on Sig1R in liver tissue using [^3^H](+)-pentazocine (Guo et al., 2013). It was shown that SCH23390 affords protection against pinacolylmethylphosphonofluoridate-evoked electrical and motor seizure activity by inhibiting D_1_ receptor (Bourne and Fosbraey, 2000) but, similar to SKF83959, does not demonstrate anti-seizure activity in a PTZ-induced seizure model (Guo et al., 2015). SCH23390 is considered a highly potent D_1_ antagonist (Hyttel, 1983) and demonstrates moderately high affinity for the 5-HT_2_ and 5-HT_1C_ receptors as well (Hicks et al., 1984). In contrast, SKF38393 is a selective D_1_ agonist (Molloy and Waddington, 1984), and the *in vivo* pharmacological activity of SKF38393 is D_1_ receptor dependent (Table 1). Overall, SKF83959 and its analogs SCH22390 and SKF38393 are non-selective allosteric modulators of Sig1R.

#### SOMCL-668

SOMCL-668 has demonstrated selective allosteric modulating activity on Sig1R (Guo et al., 2015). In the [^3^H](+)-pentazocine binding assay, SOMCL-668 shifted the saturation curve toward the left and significantly decreased the dissociation constant of the radioligand (Guo et al., 2015). SOMCL-668 synergized the effect of (+)-SKF-10,047 on Sig1R dissociation from binding immunoglobulin protein, which was further confirmed by immunoprecipitation and the plasma membrane translocation assay (Wang et al., 2016). SOMCL-668 increased the phosphorylation of glycogen synthase kinase-3β (GSK3β) on the Ser^9^ epitope and potentiated the agonist (+)-SKF-10,047-increased phosphorylation of (Ser^9^)-GSK3β in the hippocampus of mice and in hippocampal neuronal HT-22 cells, which was abolished by pretreatment with the Sig1R antagonist BD-1047 or by the knockdown of Sig1R in HT-22 cells (Wang et al., 2016). SOMCL-668 also significantly potentiated (+)-SKF-10,047-stimulated neurite growth and the production of BDNF (Wang et al., 2016), and the effect was Sig1R dependent.

The selective Sig1R allosteric modulator SOMCL-668 has been tested for its potential anti-depressant activity (Wang et al., 2016). SOMCL-668 at doses of 10 and 20 mg/kg significantly decreased the immobility time of mice in the forced-swimming and tail suspension tests, and these effects were blocked by the BD-1047 (Wang et al., 2016). In addition, daily administration of SOMCL-668 at a dose of 10 mg/kg for 1 week significantly reversed the decrease in the sucrose preference in the chronic mild stress model in mice (Wang et al., 2016). SOMCL-668 at a dose of 40 mg/kg also raised the seizure threshold in the maximal electroshock seizure test and prolonged the latencies to the clonus and generalized tonic-clonic convulsions as well as the survival time in the PTZ-induced seizure model (Guo et al., 2015). In kainic acid-induced *status epilepticus* SOMCL-668 prolonged the latency to seizure, lowered the average severity of seizure and shortened the duration of seizure (Guo et al., 2015). Moreover, the Sig1R antagonist BD-1047 at a dose of 1 mg/kg abolished the anti-seizure activity of SOMCL-668, indicating that its effect was Sig1R dependent (Guo et al., 2015; Table 1). Only Sig1R has been demonstrated to be involved in the pharmacological activities of SOMCL-668; therefore SOMCL-668 is considered a selective PAM of Sig1R.

### OZP002

The oxazaphosphinane compound did not inhibit [^3^H](+)-pentazocine binding to sigma sites, but it did induce Sig1R receptor-like effects *in vivo* and potentiated the behavioral efficacy of Sig1R agonists. OZP002 had an antidepressant effect in the forced swimming test at 10 mg/kg and potentiated the effect of the Sig1R agonist igmesine at 5 mg/kg. These effects were blocked by coinjection of the Sig1R antagonist NE-100 or were absent in Sig1R-knock-out mice (Maurice et al., 2017). OZP002 prevented scopolamine-induced learning and memory impairments (spontaneous alternation or passive avoidance) at 0.1–0.3 mg/kg, and these effects were blocked not only by NE-100 but also, interestingly, by the α7 nicotinic acetylcholine receptor antagonist methyllycaconitine (Maurice et al., 2017). Moreover, the compound was analyzed for its neuroprotective activity in a pharmacological model of Alzheimer's disease, induced in mice by intracerebroventricular injection of oligomerized amyloid Aß_25−35_ peptide, and in APP_Swe_ mice after a 2-months treatment. OZP002 prevented learning deficits in both models and decreased the expression levels of several markers of apoptosis, neuroinflammation and oxidative stress (Maurice et al., 2017). The drug therefore clearly acted as a Sig1R positive modulator and showed *in vivo* pharmacological activity closely related to that of Sig1R agonists, combining pharmacological efficacy, selectivity and therapeutic safety.

### Fenfluramine (ZX008)

Fenfluramine has recently been shown in 2 phase 3 studies to have anti-convulsant activity in a childhood epilepsy condition, Dravet syndrome (Polster, 2018). It acts as a 5-HT agonist by interacting with 5-HT_1D_, 5-HT_2A_, and 5-HT_2C_ receptors (Sourbron et al., 2016). The drug also interacts with Sig1R at nanomolar concentrations (Martin et al., 2017). In the Sig1R/BiP dissociation assay, it did not promote Sig1R/BiP dissociation but rather potentiated the effect of the Sig1R agonist PRE-084, suggesting a positive modulatory activity. The (+)-isomer of fenfluramine but not (-)-fenfluramine attenuated dizocilpine-induced deficits in a similar manner as PRE-084 (Maurice et al., 2018). It must be noted that the racemate and individual isomers of fenfluramine-related compound norfenfluramine did not affect dizocilpine-amnesia but rather prevented the PRE-084 effect, suggesting Sig1R antagonism. Combination between low doses of fenfluramine or (+)-fenfluramine and PRE-084 followed by calculation of combination indexes (Zhao et al., 2010; Maurice, 2016) showed that most combinations led to synergistic effects. Fenfluramine, as well as its active isomer (+)-fenfluramine, behaved *in vitro* and *in vivo* as Sig1R positive modulators. The drug is therefore a mixed 5-HT releaser/5-HT agonist and Sig1R modulator. Moreover, as the neuromodulatory role of Sig1R on neurotransmitter release, receptor activation and regulation of numerous ionophores is well-described, Sig1R modulation must be considered in the potential mechanism of action of its anti-convulsant activity demonstrated for Dravet syndrome (Polster, 2018).

## Comparison of the Effects of Allosteric Sig1R Modulators

### Stereochemistry of Allosteric Sig1R Modulators

**Figure 3:**
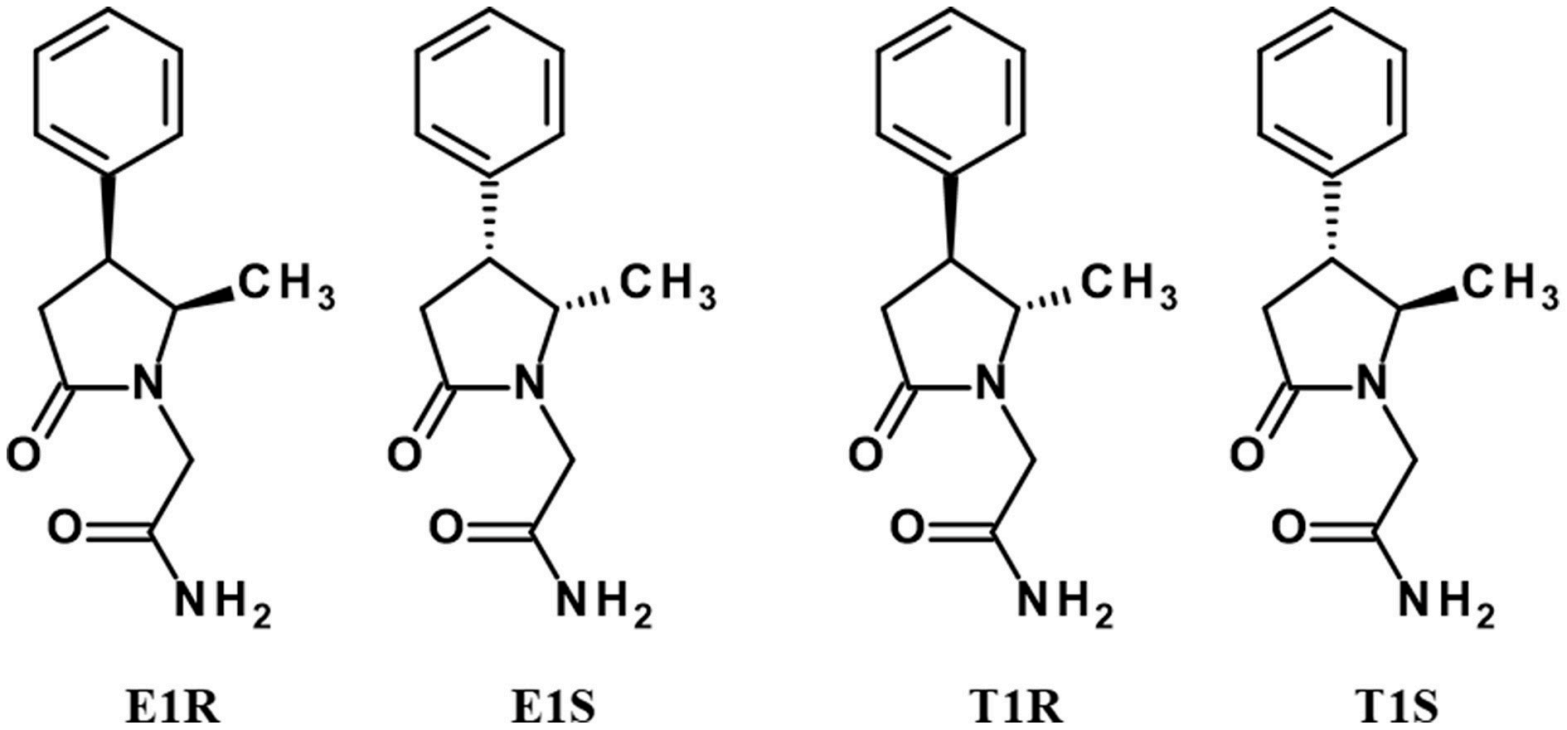
Stereoisomers of methylphenylpiracetam.

A number of Sig1R ligands contains chiral centers in their molecular structures, which indicates that compounds are optically active and that there are possibly different stereoisomers of the same molecule, which also corresponds to different pharmacological activities for the same molecules. Geometric isomers of ropizine in *cis* and *trans* forms are possible, but the molecule of ropizine is not optically active. Additionally, phenytoin is not an optically active compound due to the lack of chiral atoms in the molecular structure. Methylphenylpiracetam is a complex molecule in terms of its stereochemistry. There are two chiral centers in the molecular structure of methylphenylpiracetam; therefore, it is possible to isolate four individual stereoisomers, which are denoted E1R, T1R, E1S, and T1S (reviewed in Veinberg et al., 2015; Figure 3).

**Figure 4:**
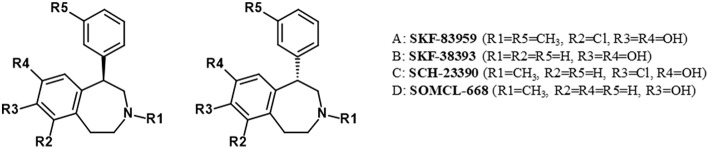
Enantiomers of benzazepine analogs.

SKF83959 is a racemate that consists of the R-(+)- and S-(–)-enantiomers MCL-202 and MCL-201, respectively (Desai et al., 2007). The R-(+)- and S-(–)-enantiomers are also possible for SKF38393, SCH23390, and SOMCL-668 (Figure 4).

All stereoisomers of methylphenylpiracetam are known to act as PAMs of Sig1R (Veinberg et al., 2013). In an *ex vivo* test the enantiomers with the R-configuration at the C-4 chiral center in the 2-pyrrolidone ring (E1R and T1R) were found to be more effective PAMs of Sig1R than their optical antipodes (Veinberg et al., 2013), showing that some stereo selectivity is possible also for allosteric Sig1R modulators.

The Sig1R modulatory activity of changes in the activity of Sig1R ligands induced by different enantiomers of benzazepine derivatives has not been compared. It would be interesting to compare the allosteric modulatory activity on Sig1R regarding the stereochemical properties of benzazepine derivatives. For example, it has been shown that the D_1_ receptor binding site favors the R-enantiomers of the D_1_ receptor-selective benzazepines over the corresponding S-enantiomers (Kebabian, 1988), which is true for both the D_1_ agonist SKF38393 and the D_1_ antagonist SCH23390 (Kebabian, 1988). In addition, the stereo selectivity of the R-isomer of SCH23390 for blockade of D_1_ receptors *in vivo* has been demonstrated (Ongini et al., 1987). In the case of benzazepine derivatives, Sig1R selectivity over dopamine receptors could depend on the pharmacological activity of individual isomers. However, this dependence has not been demonstrated so far.

The structure-activity relationship for fenfluramine is rather clear since the positive modulator activity of the drug originates from only the dextrorotatory isomer (+)-fenfluramine. In all behavioral responses analyzed, the racemate behaved as (+)-fenfluramine, suggesting chiral binding of the drug to the yet unidentified, modulatory binding site on Sig1R.

### *In vitro* Effects

No screening assay is available for allosteric modulators of Sig1R. Therefore, different *in vitro* assays and *in vivo* pharmacological approaches have been used to describe and validate the activity of allosteric Sig1R modulators. For comparison, the Sig1R-related *in vitro* activities of allosteric Sig1R modulators are summarized in Table 2.

**Table 2:** The comparison of *in vitro* allosteric effects of Sig1R modulators.

**Compound**	**[μM]**	**Activity**	**Sig1R ligand**	**Tissues/cells (species)**	**References**
Phenytoin	10–100	Increases the binding	[^3^H]DM	Brain tissues (guinea pig)	Craviso and Musacchio, 1983; Musacchio et al., 1988, 1989
	10–100	Increases the binding	[^3^H](+)-3-PPP	Brain tissues (guinea pig)	Musacchio et al., 1989
	300	Increases the binding	[^3^H](+)-SKF-10,047	Brain tissues (guinea pig)	Karbon et al., 1991
	300	Increases the binding	[^3^H](+)-SKF-10,047	Liver tissues (rat)	McCann and Su, 1991
	0.1–250	Increases the binding	[^3^H](+)-Pentazocine	Brain tissues (guinea pig)	DeHaven-Hudkins et al., 1993; Cobos et al., 2005, 2006
	100–10,000	Increases the binding	[^3^H](+)-Pentazocine	Brain tissues (rat)	Guo et al., 2013
	1,000	Increases the binding affinity of dm	[^3^H](+)-Pentazocine	Lung tissues (mice)	Lever et al., 2015
	100	No effect	[^3^H]DM	Liver tissues (guinea pig)	Craviso and Musacchio, 1983
	0.0001–100	No effect	[^3^H](+)-Pentazocine	Brain tissues (rat)	Zvejniece et al., 2014
	1–10,000	No effect	[^3^H](+)-Pentazocine	Liver tissues (rat)	Guo et al., 2013
	10, 100	No effect	[^3^H](+)-Pentazocine	Constructed HEK293 cells	
	10, 100/300	No effect	[^3^H]DTG	Brain tissues (guinea pig/ rat)	Karbon et al., 1991; Guo et al., 2013
	0.1–250	Decreases the binding	[^3^H]NE-100	Brain tissues (guinea pig)	Cobos et al., 2006
	10, 100	No effect	[^3^H]Progesterone	Brain and liver tissues (rat)	Guo et al., 2013
Ropizine (SC-13504)	0.1–10	Increases the binding	[^3^H]DM	Brain tissues (guinea pig)	Musacchio et al., 1988, 1989; Klein and Musacchio, 1992
	0.1–10	Increases the binding	[^3^H](+)-3-PPP	Brain tissues (guinea pig)	Musacchio et al., 1989; Klein and Musacchio, 1990, 1992
SKF83959	0.1–100	Increases the binding	[^3^H](+)-Pentazocine	Brain and liver tissues (rat)	Guo et al., 2013
	0.1–10	Increases the binding affinity of DHEA	[^3^H](+)-Pentazocine	Brain tissues (rat)	Wu et al., 2015
	0.1	Enhances the anti-inflammatory effect on LPS induced inflammation	DHEA	Microglial BV-2 cells (mice)	
	1	Enhances the anti-inflammatory effect on LPS induced inflammation	PRE-084	Microglial BV-2 cells (mice)	
	10, 100	No effect	[^3^H](+)-Pentazocine	Constructed HEK293 cells	Guo et al., 2013
	10, 100	No effect	[^3^H]Progesterone	Brain and liver tissues (rat)	
	10, 100	No effect	[^3^H]DTG	Brain and liver tissues (rat)	
SCH23390	0.1–100	Increases the binding	[^3^H](+)-Pentazocine	Liver tissues (rat)	Guo et al., 2013
	0.001–100	No effect	[^3^H](+)-Pentazocine	Brain tissues (rat)	
	10, 100	No effect	[^3^H](+)-Pentazocine	Constructed HEK293 cells	
	10, 100	No effect	[^3^H]Progesterone	Brain and liver tissues (rat)	
	10, 100	No effect	[^3^H]DTG	Brain and liver tissues (rat)	
SKF38393	0.1–100	Increases the binding	[^3^H](+)-Pentazocine	Liver tissues (rat)	Guo et al., 2013
	0.001–100	No effect	[^3^H](+)-Pentazocine	Brain tissues (rat)	
	10, 100	No effect	[^3^H](+)-Pentazocine	Constructed HEK293 cells	
	10, 100	No effect	[^3^H]Progesterone	Brain and liver tissues (rat)	
	10, 100	No effect	[^3^H]DTG	Brain and liver tissues (rat)	
SOMCL-668	100	Increases the binding	[^3^H](+)-Pentazocine	Brain tissues (rat)	Guo et al., 2015
	10	Enhances the translocation of Sig1R from BiP	(+)-SKF-10,047	Hippocampal neuronal HT-22 cells (mice)	Wang et al., 2016
	10	Enhances stimulated neurite growth and BDNF secretion	(+)-SKF-10,047	Primary cortical/hippocampal neurons (mice)	
E1R	10	Increases the binding	[^3^H]DTG	Jurkat cells (human)	Zvejniece et al., 2014
	0.0001–100	No effect	[^3^H](+)-Pentazocine	Brain tissues (rat)	
	10	Enhances the activity on electrically stimulated contractions	PRE-084	*vasa deferentia* (rat)	Veinberg et al., 2013; Zvejniece et al., 2014
	10	No effect	PB-28	*vasa deferentia* (rat)	Zvejniece et al., 2014
	10	Enhances the activity on the BDK-induced [Ca^2+^]_i_ increase	PRE-084	NG108-15 cells (rat and mice)	
OZP002	1	No effect	[^3^H]DTG	Jurkat cells (human)	Maurice et al., 2017
	1–30	Increases the binding	[^3^H](+)-Pentazocine	Jurkat cells (human)	
Fenfluramine (ZX008)	0.0001–100	Inhibits the binding	[^3^H]DTG	Jurkat cells (human)	Martin et al., 2017
	0.0001–100	Inhibits the binding	[^3^H](+)-Pentazocine	Jurkat cells (human)	Maurice et al., 2018
	3–10	Enhances agonist-induced activity on electrically stimulated contractions	(+)-SKF-10,047	*vasa deferentia* (guinea pig)	
	1–10	Enhances the translocation of Sig1R from BiP	PRE-084	CHO cells	

The allosteric modulatory activity of phenytoin has previously been described in rat and guinea pig brains (DeHaven-Hudkins et al., 1993; Cobos et al., 2006; Guo et al., 2013), rat livers (McCann and Su, 1991), and mice lung tissues (Lever et al., 2015). In turn, it has been shown that phenytoin could modulate Sig1R ligand binding in rat brain tissue but not in rat liver tissue (Guo et al., 2013, Table 2). For example, SCH23390 and SKF38393 modulated the binding of [^3^H](+)-pentazocine only in liver tissues, while no detectable effects were observed in brain tissues (Guo et al., 2013). In the same study, SKF83959 was shown to allosterically modulate the binding of [^3^H](+)-pentazocine in both rat brain and liver tissues (Guo et al., 2013). The different effects of these Sig1R allosteric modulators in rat and guinea pig brain tissues have been described previously and could be explained by the variation in the size of the binding site between the two species (Klein and Musacchio, 1992). It seems that not only different species but also different tissues from the same animal species can respond differently to allosteric Sig1R modulators. SKF83959 and its analogs failed to change the binding activity of [^3^H](+)-pentazocine at Sig1R in human embryonic kidney (HEK)293 cells that stably expressed the Sig1R (Guo et al., 2013). The lack of binding modulatory activity of [^3^H](+)-pentazocine in HEK293 cells was also observed for phenytoin (Guo et al., 2013). Although [^3^H](+)-pentazocine displayed similar affinity for Sig1R in transfected HEK293 cells and rat brain tissues, the absence of allosteric modulation of Sig1R in the constructed system *in vitro* could be attributed to differences in Sig1R structure, cellular contents, or auxiliary proteins (Guo et al., 2013), which should be taken into account when studying mechanisms of Sig1R *in vitro*.

Above described issues rise number of questions in the understanding of molecular mechanisms of allosteric Sig1R modulators. Why allosteric modulatory activity of compounds has been observed in one type of tissues and/or animal species but not in the others? Is that because of the structure differences between compounds? Could it be because of different Sig1R structures, conformation states or even tissue specific Sig1R subtypes? Are there species specific Sig1R? It should be noted that some observed allosteric effects on Sig1R were shown after administration of allosteric modulator in high concentrations (Table 2). Thereby, how Sig1R-specific is the activity of allosteric modulators? Are there other proteins involved in the activity of compounds? What is the influence of endogenous compounds? Future studies must be focused to answer these questions. Nevertheless, it is clear that allosteric Sig1R modulators could be used as pharmacological tools to increase the global understanding of the physiological function of Sig1R and its interaction with ligands. Also the Sig1R-related *in vivo* activities of allosteric Sig1R modulators are very promising to reach clinical advantages.

### Anti-seizure Activity

All allosteric Sig1R modulators demonstrate anti-seizure activity. However, the anti-seizure activity is not associated directly with Sig1R for most of allosteric Sig1R modulators (Table 3).

**Table 3:** Sig1R-dependent anti-seizure activity of allosteric Sig1R modulators.

**Compound**	**Dose, mg/kg**	**Seizure model**	**Effects**	**References**
SKF83959	2	Maximal electroshock seizure threshold test	No significant effect	Guo et al., 2015
	10		Increased the seizure threshold	
	20			
	40			
	2	PTZ (80 mg/kg, s.c.)	No significant effect	
	10		No significant effect	
	20		Prolonged the latencies of clonic and generalized clonic-tonic seizures, survival time, and significantly lowered seizure scores	
	40			
	2	Kainic acid (30 mg/kg, i.p.)	No significant effect	
	10		No significant effect	
	20		No significant effect	
	40		Significantly reduced seizure incidence, prolonged the latency to seizures, and shortened the duration of seizures	
SOMCL-668	40	Maximal electroshock seizure threshold test	Increased the seizure threshold	Guo et al., 2015
	40	PTZ (80 mg/kg, s.c.)	Prolonged the latency time to the generalized clonic-tonic seizures and survival time	
	40	Kainic acid (30 mg/kg, i.p.)	Prolonged the latency time and shortened the duration of seizures	
E1R	10	PTZ (i.v. infusion)	Increased the threshold for clonic and tonic seizures	Vavers et al., 2017
	50			
	10	BIC (i.v. infusion)	No significant effect	
	50		Increased the threshold for clonic and tonic seizures	
	75	NE-100 (75 mg/kg, i.p.)^*^	Significantly reduced generalized seizure count and average behavioral score	
Fenfluramine (ZX008)	Maximum Tolerable Concentration	Homozygous *scn1a* mutant zebrafish larvae	Decreased epileptiform activity	Sourbron et al., 2017
	3 nmol	NMDA (i.c.v. administration)	Reduced rearing, hypermotility-circling, clonic convulsions, tonic seizures, and death	Rodríguez-Muñoz et al., 2018
	500 μM	Low Mg^2+^	Blockade of early and late epileptiform activity	Gentsch et al., 2000

* *NE-100 induced seizures in 100% of animals at a dose of 75 mg/kg (Vavers et al., 2017). Phenytoin, ropizine, SCH23390, and SKF38393 are not included in the table because the seizure modulating activities of these compounds are not shown to be significantly blocked by the selective Sig1R ligands*.

It was demonstrated that the anti-seizure effect of E1R, SOMCL-668, and SKF83959 was blocked by the Sig1R selective antagonists NE-100 and BD-1047 (Guo et al., 2015; Vavers et al., 2017), while the anti-seizure effect of phenytoin was not blocked by BD-1047 (Guo et al., 2015). Moreover, anti-seizure effect of phenytoin is related to the inhibition of voltage-gated sodium channels (Tunnicliff, 1996). At the same time, it was shown that SCH23390, an analog of SKF83959, does not affect PTZ-induced seizures (Guo et al., 2015). Rather, SCH23390 has been shown to modulate seizures evoked by the subcutaneous administration of the cholinesterase inhibitor pinacolylmethylphosphonofluoridate and this activity was demonstrated to be D_1_ receptor dependent (Bourne et al., 2001). Current knowledge indicates that not all PAMs of Sig1R possess anti-seizure activities and that the anti-seizure effects are not always regulated by Sig1R.

### Memory Improvement

Among all the positive allosteric Sig1R modulators described, E1R, OZP002, and fenfluramine showed Sig1R-dependent memory-improving effects (Zvejniece et al., 2014; Maurice et al., 2017, 2018). E1R, however, is the only modulator showing dose-dependent memory-improving activity in drug-naïve animals (Zvejniece et al., 2014). E1R and OZP002 successfully alleviated scopolamine-induced cognitive impairment in mice, as assessed using passive avoidance and spontaneous alternation tests (Zvejniece et al., 2014; Maurice et al., 2017). The effects were antagonized by the selective Sig1R antagonist NE-100, thus confirming the Sig1R modulatory activity of E1R *in vivo*. Moreover, fenfluramine showed anti-amnesic effects in mice treated with the non-competitive NMDA receptor antagonist dizocilpine (Maurice et al., 2018). This activity has long been described for Sig1R agonists (Maurice et al., 1994a,b) and is usually considered a potential activity test confirming the Sig1R agonist or antagonist activity of selective drugs. Finally, OZP002 showed anti-amnesic effects against learning deficits induced by amyloid Aβ_25−35_ peptide, a pharmacological model of symptomatic efficacy in Alzheimer's diease (Maurice et al., 1998). Some memory improvement activities have also been demonstrated for benzazepines SKF83959 and SKF38393. For example, administration of SKF83959 significantly improved scopolamine-induced memory impairments in the passive avoidance task, spontaneous alternation test, and place learning task in the Morris water maze task in mice (Sheng et al., 2018; Table 1). SKF38393 has been shown to improve temporal order memory performance in maternally deprived rats (Lejeune et al., 2013; Table 1). However, the effects of SKF83959 and SKF38393 on memory are dopamine receptor-mediated, and the involvement of Sig1R in the memory improvement of these compounds has not been demonstrated. In contrast to E1R treatment, treatment with phenytoin triggered memory impairment during the passive avoidance task (Reeta et al., 2009). Treatment of epilepsy with phenytoin has been shown to induce learning and memory deficits in patients as well (Mishra and Goel, 2015). Sig1R-related memory-improving activity could be specific for selective allosteric Sig1R modulators such as E1R and SOMCL-668. However, it has not been demonstrated that SOMCL-668 improves memory and cognition through a Sig1R-related pathway. Therefore, to date, the Sig1R-related memory-improving activity is specific only for E1R, OZP002, and fenfluramine.

### Anti-depressant Activity

To date, SOMCL-668, OZP002 and fenfluramine have shown anti-depressant activity, which has been demonstrated to be Sig1R specific. The anti-depressant activity of SKF-83959 was shown to be regulated through other mechanisms (Table 1). It has been described that stereoisomers of methylphenylpiracetam, including E1R, did not induce any significant effects on the depressive condition in mice (Vavers et al., 2015). In the case of phenytoin, major depression as a complication of phenytoin intoxication has been described (Levkovitch et al., 1993). Therefore, anti-depressant activity seems to be shared by but not specific to allosteric modulators of Sig1R.

## Allosteric Modulation of Sig1R

There are no clearly defined molecular mechanisms that could fully describe the function of Sig1R and the activity of Sig1R ligands. Thus, it is more difficult to describe the activity of allosteric modulators, which do not compete with orthosteric Sig1R ligands for binding in the active site of Sig1R but rather enhance the activity of Sig1R agonists. The crystal structure of Sig1R shows that the ligand-binding domain in the protein is highly conserved, and how ligands enter and exit this site remains unclear (Schmidt et al., 2016). The binding site for allosteric Sig1R modulators is probably located outside the orthosteric ligand-binding domain. Since allosteric modulators are compounds that induce a conformational change within the protein structure, PAMs should reorganize the Sig1R protein in a way that would allow agonists to freely enter the ligand-binding site. It has been discussed previously that phenytoin might induce a conformational change in the Sig1R and thus enhance the affinity of the orthosteric ligand [^3^H](+)-pentazocine for its binding site on Sig1R (Cobos et al., 2006), which fits classic description of the activity of allosteric modulators (**Figure 6**). However, it is not clear how allosteric modulators of Sig1R can distinguish between agonists and antagonists and then selectively enhance the activity of agonists, even though the agonists and antagonists sometimes contain the same structural moieties.

**Figure 5:**
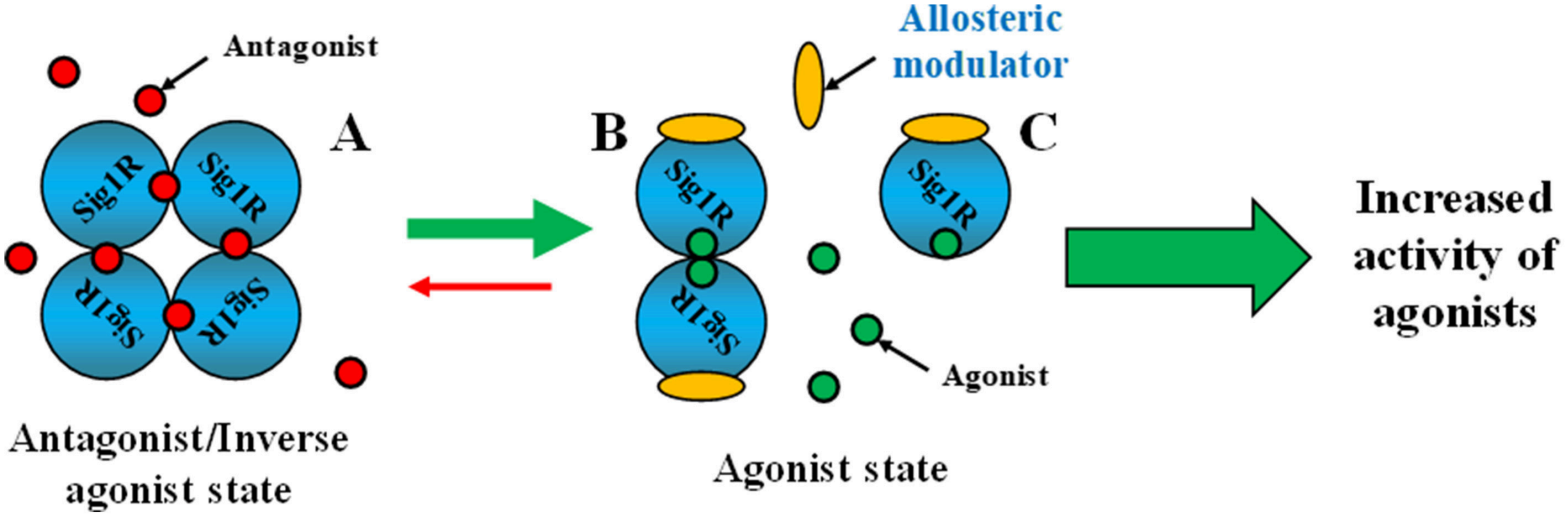
Stabilization of the agonist state of Sig1R by PAMs. A model showing possible mechanisms of Sig1R ligand activity. **(A)** Oligomeric form. For the agonist state, **(B,C)** represent the dimeric and monomeric forms of Sig1R, respectively. This Figure has been modified from (Chu and Ruoho, 2016).

**Figure 6:**
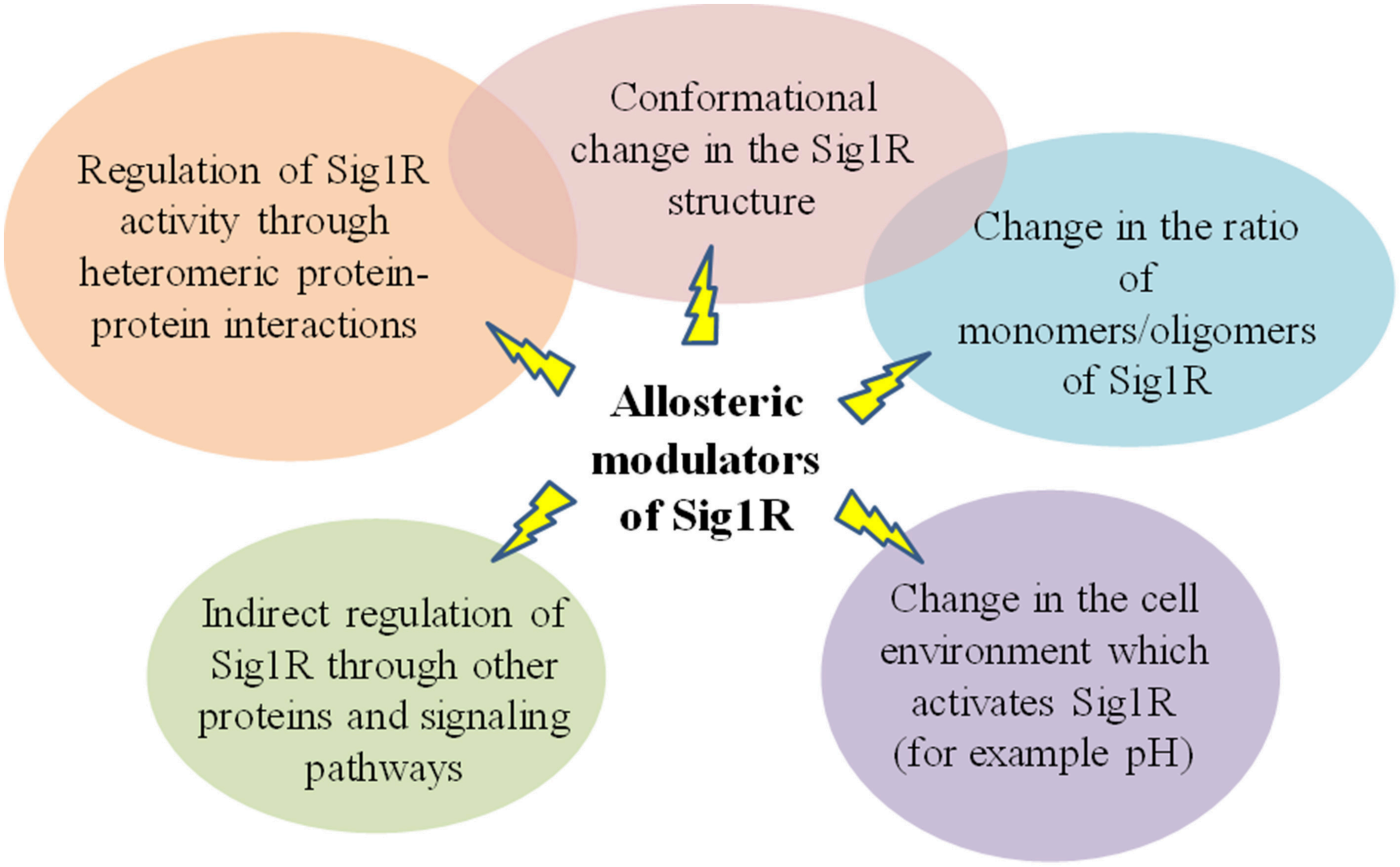
Summary of possible mechanisms of allosteric modulators of Sig1R.

Classical models for allosteric modulation might not be attributed to allosteric modulation of Sig1R. Several previous experiments support the conclusion that in ligand binding, Sig1R functions as an oligomer (Schuster et al., 1995; Chu et al., 2013; Gromek et al., 2014). Therefore, oligomerization is a key functional property of the Sig1R that may be linked to ligand efficacy [Schmidt et al., 2016; reviewed in Chu and Ruoho (2016)] and could explain how ligands can regulate the activity of Sig1R (**Figure 6**). Sig1R ligands may regulate the activity of the receptor interaction with client proteins by altering the oligomeric/monomeric receptor ratio and favoring the oligomeric states (Mishra et al., 2015). It has been shown that the Sig1R agonist (+)-pentazocine increased the relative ratio of dimers and monomers, while the inhibitor haloperidol increased the incidence of higher oligomeric forms (Chu and Ruoho, 2016). This observation indicates that higher oligomeric forms of Sig1R might be functionally inactive (Figure 5A). Since all Sig1R allosteric modulators known thus far are PAMs and enhance the activity of Sig1R agonists, they might modulate Sig1R by stabilizing the agonist state of the receptor, providing an increase in the dimeric (Figure 5B) and/or monomeric (Figure 5C) protein forms.

Sig1R has already been described as a chaperone that modulates other receptor systems through heteromeric protein-protein interactions (Navarro et al., 2013; Pabba, 2013; Moreno et al., 2014; Su et al., 2016). Therefore, it is possible that heteromeric complexes formed by Sig1R and its target proteins could be regulated by allosteric modulators of Sig1R (Figure 6). Allosteric Sig1R modulators could serve as a bridge between Sig1R and its target proteins. The “molecular glue” concept was first discovered by observing the mechanism of action of natural products that promote immunomodulatory ternary complex formation (Che et al., 2018), and recently, some compounds of synthetic origin have been reported to induce novel protein-protein interactions [reviewed in (Che et al., 2018)]. The “molecular glue” principle could be attributed not only to heteromeric protein-protein interactions but also to homomeric Sig1R interactions and could explain how allosteric modulators could increase the number of Sig1R in the agonist state conformation, thus increasing the activity of Sig1R agonists.

It has been shown that compounds that bind to allosteric site of protein may activate the receptor in the absence of agonist (Schwartz and Holst, 2007). This condition, known as allosteric agonism, could explain the direct pharmacological effects of allosteric modulators of Sig1R observed both *in vitro* and *in vivo* (Zvejniece et al., 2014; Guo et al., 2015; Wang et al., 2016; Maurice et al., 2017, 2018). “Superagnonism” or ago-allosteric modulation also could be one of the possible descriptions of the modulatory activity of allosteric Sig1R ligands. An ago-allosteric modulator acts as both an agonist and an enhancer of agonist potency and provides “superagonism,” which would result in an efficacy >100% (Schwartz and Holst, 2007). One example of ago-allosteric modulatory activity could be the published *in vitro* effects of E1R (Zvejniece et al., 2014). It was shown that both selective Sig1R agonist PRE-084 and allosteric modulator E1R increased the BDK-induced [Ca^2+^]_i_ increase, while the combination of both compounds resulted in an even more pronounced cellular response (Zvejniece et al., 2014). The similar “superagonism” could also be attributed to SOMCL-668. Both the allosteric Sig1R modulator SOMCL-668 and Sig1R agonist (+)-SKF-10,047 increased P(Ser^9^)-GSK-3β in hippocampal neuronal HT-22 cells, while the (+)-SKF-10,047-increased phosphorylation was potentiated by SOMCL-668 (Wang et al., 2016). While “superagonism” could be attributed only to some *in vitro* effects of E1R and SOMCL-668, it cannot be concluded that allosteric Sig1R modulators are superagonists. In addition, the direct pharmacological effects of allosteric Sig1R modulators could be explained by the presence of a possible endogenous agonists of Sig1R. N,N-dimethyltryptamine is considered as one of the most active endogenous Sig1R agonists identified thus far (Fontanilla et al., 2009; Su et al., 2009). Very recently, it was shown that also choline acts as an endogenous ligand (Brailoiu et al., 2019). However, to date no study has demonstrated the enhancement of endogenous agonist activity by known allosteric modulators of Sig1R. The acidity or alkalinity of a studied system is another issue that should be considered when evaluating possible mechanisms of allosteric Sig1R modulators (Figure 6) because it has been shown that pH by itself changes the activity of Sig1R ligands. Binding of ligands to Sig1R is pH dependent and is enhanced at higher pH (Largent et al., 1987). [^3^H](+)-Pentazocine binding to C6 glioma cell membranes was progressively increased at pH = 8.0 compared to buffer at pH = 7.0 and pH = 7.4 (Vilner et al., 1995). In addition, it was discovered that the pH of the medium markedly affected the activity of the Sig1R compounds in C6 glioma cells (Vilner et al., 1995). Increasing the pH from the range 7.2–7.4 to 8.3–8.5 caused a marked leftward shift in the dose curves for all active Sig1R compounds, while lowering the pH generally decreased the ability of sigma compounds to change cell morphology (Vilner et al., 1995). As it is known, all of the allosteric Sig1R modulators induce the same effect, which means an increase in the binding and activity of Sig1R ligands. Can allosteric modulators of Sig1R change the intracellular pH? Interestingly, it was previously demonstrated that a phenytoin-induced increase in the binding affinity of [^3^H]DM to brain homogenate is more marked at pH = 7.4 than at pH = 8.3 (Musacchio et al., 1988). Phenytoin is a weak acid with a pKa of approximately pH = 8.3. Therefore, at pH = 7.4 the concentration of unionized phenytoin is almost doubled, which may explain why phenytoin is much more potent at pH 7.4 (Musacchio et al., 1988). This finding demonstrates that pH plays a significant role in the process that determines the interaction between Sig1R ligands and receptors. Protonation or deprotonation of amino acids due to different pH could explain the change in the structure of Sig1R and therefore the change in the binding affinity of ligands to Sig1R. What is the role of allosteric modulators in these processes? In a number of structure-activity relationship studies, only agonists or antagonists have been used for evaluation of possible ligand-protein interactions. Additionally, no NMR data are available where allosteric modulators have been used to describe their molecular interaction with Sig1R. Most likely, three or more component systems (allosteric modulator-Sig1R-agonist and/or antagonist) must be applied to understand the interaction with Sig1R at the protein level. Detailed studies on allosteric Sig1R modulators at the protein level would be necessary to increase the global understanding of the interaction with the receptor and activity of Sig1R ligands.

## Key Issues

### Characterization of Binding Site

The binding site(s) for Sig1R allosteric modulators is(are) not identified. The binding dynamics in the Sig1R for allosteric compounds must be confirmed and characterized.

### Screening System

No screening assay is available for allosteric modulators of Sig1R and must be developed and validated. Notably, modulation of the agonist-induced dissociation of Sig1R from BiP *in vitro* must be confirmed with all already identified modulators.

### Stereoselectivity

The use of enantiomerically pure compounds is necessary to provide detailed evaluation of the *in vitro* and *in vivo* effects of allosteric Sig1R modulators. Comparison of all allosteric Sig1R modulators in the same experimental setup should be performed.

### *In vivo* Proof of the Sig1R-Related Mechanism of Action

There is a lack of *in vivo* studies combining Sig1R agonists and allosteric Sig1R modulators to confirm the allosteric modulatory activity of a compound at Sig1R. *In vitro* concentrations and therapeutic doses *in vivo* should be comparable.

## Conclusion

Allosteric pharmacology, that is, the design of drugs targeting sites topographically different from the orthosteric ligand binding site, is innovative approach to drug discovery. Allosteric modulation of Sig1R is an emerging and important target for designing novel drugs. A number of issues must be answered regarding the mechanisms of action and possible clinical applications of allosteric Sig1R modulators. More detailed classification of Sig1R ligands is inevitably necessary, and the development of new screening assay methods focused on ligand characterization is highly important to advance the understanding of the role of Sig1R and its allosteric modulators. Nevertheless, during the last 6 years, a large step forward in the understanding of allosteric modulation of Sig1R has been made. The first selective allosteric Sig1R modulators have been identified, which offer a basis for the discovery of novel and selective Sig1R compounds and increase knowledge of the impact of Sig1R regulation in living organisms, thereby providing novel treatment possibilities for various CNS-related diseases.

## Author Contributions

EV and LZ wrote the manuscript in consultation with TM and MD. TM provided additional information on OZP002 and fenfluramine. All authors discussed the data and commented on the manuscript. MD supervised the manuscript.

### Conflict of Interest Statement

TM is co-inventor on the patent (PCT/EP2017/060129) describing OZP002 and received consultancy fees and research contracts from Zogenix Inc. The company had no role in the writing of this review. The remaining authors declare that the research was conducted in the absence of any commercial or financial relationships that could be construed as a potential conflict of interest.
